# Orthoplastic management of Gustilo–Anderson type IIIB/C open tibial fractures: a consecutive 10-year series from China Level I trauma center

**DOI:** 10.1097/JS9.0000000000002809

**Published:** 2025-07-08

**Authors:** Yapeng Wang, Ming Zhou, Peng Wang, Jun Liu, Yunhong Ma, Gang Zhao, Yongwei Wu, Yongjun Rui

**Affiliations:** a Soochow University, Suzhou City, Jiangsu Province, China; b Department of Orthoplastic Surgery, Wuxi No. 9 People’s Hospital Affiliated to Soochow University, Wuxi, Jiangsu Province, China

**Keywords:** bone graft, Gustilo–Anderson type IIIB/C, open tibial fractures, orthoplastic approach

## Abstract

**Background:**

Research on open tibiofibular fractures is limited. We compared clinical outcomes of Gustilo**–**Anderson type IIIB/C open tibial fractures using orthopedic and orthoplastic approaches, and identified poor prognosis predictors.

**Materials and Methods:**

The clinical data of 746 patients with Gustilo**–**Anderson type IIIB/C open tibial fractures (420 and 326 treated using the orthopedic and orthoplastic approach, respectively) were retrospectively analyzed. We evaluated infection rates, nonunion incidence, arthritis incidence, number of surgeries, fracture healing time, amputation rate, wound closure duration, and Lower Extremity Functional Scale (LEFS) score, and identified risk factors affecting prognosis.

**Results:**

Significant differences in infection rates (deep infection, 8.0% vs. 29%; superficial infection, 8.0% vs. 41%; all *P*<0.001), nonunion incidence (11% vs. 2.5%, *P*<0.001), arthritis incidence (27% vs. 2.1%, *P* < 0.001), number of surgeries (4.6 ± 1.07 vs. 10.7 ± 3.33, *P* < 0.001), and wound coverage time (11.27 ± 5.14 vs. 3.98 ± 1.98, *P* < 0.001) were observed between the groups. LEFS scores from 3 to 24 months after injury were higher in the orthoplastic group. No difference in fracture healing time was observed (7.0 ± 1.99 vs. 7.0 ± 1.98, *P* = 0.987). The orthoplastic group required a lower amount of bone graft when using intramedullary nail fixation (6.8 ± 1.42 vs. 19.0 ± 2.88, *P* < 0.001). Smoking (odds ratio [OR], 0.24 for nonunion; 95% confidence interval [CI], 2.29–5.34; *P* = 0.008 and OR, 0.26 for deep infection; 95% CI, 0.10–0.71; *P* = 0.009), bone cement block formation (OR, 1.54; 95% CI, 2.06–4.73; *P* = 0.007), and local antibiotic use (OR, 4.89; 95% CI, 1.93–12.37; *P* < 0.001) were predictors of poor prognosis.

**Conclusion:**

The orthoplastic approach offers advantages in the treatment of Gustilo**–**Anderson type IIIB/C open tibial fractures. Smoking should be avoided, bone cement block molding should be actively adopted, and systemic and local antibiotics should be administered as early as possible. The Flap and Open Reduction Internal Fixation and Masquelet technique reduces the amount of bone graft without increasing deep infection risk.

## Introduction

Open tibial fractures represent severe injuries frequently encountered in orthopedic practice, comprising approximately 45% of all open long bone fractures. These fractures are predominantly the result of high-energy trauma, such as traffic accidents and falls from heights^[1,2]^. Their distinctive characteristics include inadequate soft tissue coverage and blood supply in the anteromedial aspect of the tibia, rendering them particularly vulnerable to bone defects, soft tissue injuries, and contamination. Consequently, they present a significantly higher risk of complications than closed fractures^[3]^. According to the Gustilo classification, type IIIB/C open tibial fractures are commonly associated with severe bone and soft tissue defects and pose challenges for treatment^[4]^.

**HIGHLIGHTS**

- Orthoplastic method cuts risks of complications in type IIIB/C tibial fractures.
- Flap + ORIF and Masquelet technique curbs bone grafting and deep infections.
- Smoking, cement block formation and local antibiotic use predict poor prognosis.

The traditional orthopedic approach primarily emphasizes repeated debridement, basic external fixation of bones, and negative-pressure suction for managing soft tissue defects. However, a systematic strategy for soft tissue repair is lacking, resulting in a reported deep infection rate of 23.1%^[5]^. Furthermore, long-term external fixation has inadequate strength, hinders early functional rehabilitation, and increases the risk of pin-tract infections^[6]^. Prolonged external fixation not only extends the overall treatment duration but also heightens the psychological and financial burden on patients. This approach may lead to malunion of the fracture ends, adversely affecting patient prognosis and functional recovery^[7]^. Additionally, delayed fixation owing to soft tissue scarring significantly complicates subsequent internal fixation surgery and increases the risk of intraoperative vascular and nerve injuries^[8]^.

In 2009, the British Orthopaedic Association proposed an orthoplastic approach for the management of open lower limb fractures, which was collaboratively implemented by orthopedic and plastic surgery departments. This approach emphasizes early and thorough debridement, fracture fixation, and soft tissue coverage to reduce the infection risk, promote fracture healing, and enhance functional recovery in patients^[9]^. In 2015, Mundi *et al* published an update on treatment guidelines for open tibial fractures^[10]^. In the United Kingdom, the management of open fractures is governed by the British Orthopaedic Association Standards for Trauma 4. Although considerable research has been conducted on orthoplastic treatment for open tibial fractures in recent years, most studies are limited to single-center retrospective analyses (Level III evidence),^[11–14]^ lacking systematic comparisons of clinical outcomes and complications between the two approaches. Furthermore, definitive fixation methods for fractures and the most appropriate timing for wound coverage have not been defined, and the factors influencing the clinical outcomes of orthoplastic procedures remain unclear^[15,16]^. Additionally, there is significant variation in treatment strategies among different practitioners and hospitals. As is the case in many developing countries, orthopedic methods have long been the primary approach for treating severe lower extremity trauma in mainland China. Notably, a considerable proportion of orthopedic surgeons in China possess both fixation and microsurgery skills, which distinguishes them from their counterparts in other developed countries. Previous research on open tibiofibular fractures has been limited, often owing to small sample size or lack of controlled studies. We conducted a retrospective controlled analysis using an existing database, benefiting from the fact that all the data were prospectively collected by our team.

We aimed to (1) compare the clinical outcomes of the orthopedic and orthoplastic approaches, (2) identify the risk factors associated with poor prognosis, and (3) optimize the orthoplastic approach for severe trauma based on our clinical experience.

## Materials and methods

### Data source and participants

The data were sourced from the Comprehensive Management of Patients with Open Fractures database, which is a carefully updated database containing information on all inpatients with open fractures at our hospital. The demographic characteristics, injury information, and surgical details of the patients were prospectively collected by trained research assistants and validated by the attending surgeon. Outcome data and complications during regular postoperative follow-up were prospectively recorded.

The use of the database was approved by our Institutional Ethics Committee (KS2024026). The study followed the principles of the Helsinki Declaration, and we report our research in accordance with the STROCSS criteria^[17]^. The requirement for informed consent was waived because of the retrospective nature of this study. All the data collected from patients were recorded anonymously to protect their privacy. This study was registered in the Chinese Clinical Trial Registry (ChiCTR2200057118).

We retrospectively reviewed the prospective database of patients diagnosed with Gustilo**–**Anderson type IIIB/C open tibial fractures between January 2007 and December 2022. The orthopedic approach was used before December 2012, whereas the orthoplastic approach was adopted after that date. The inclusion criteria were as follows: (1) Gustilo**–**Anderson type IIIB/C open tibial fracture, (2) age >18 years, and (3) follow-up for >26 months. The exclusion criteria were as follows: (1) patients who had not undergone emergency surgery at our hospital according to the orthopedic or orthoplastic approach; (2) emergency amputees; (3) patients with a history of mental illness; (4) patients with a history of central nervous system diseases such as encephalitis or brain tumors; (5) patients who were unconscious upon admission, including those in a coma; and (6) patients with incomplete follow-up data. Baseline data, radiological findings, and clinical outcomes were reviewed and analyzed.

### Surgical procedure

All surgical procedures were performed by trained surgeons.

**Orthopaedic approach**: Surgery was divided into four stages, with the first stage being the emergency stage. The recommended regimen includes cefuroxime combined with aminoglycoside antibiotics, or clindamycin for patients with a β-lactam allergy. The wound was rinsed with 9 L of isotonic sodium chloride solution in the operating room and thoroughly debrided by the orthopedic surgeons to remove non-viable and contaminated tissues. All free tibial fragments (inactive bone) were removed, and the length of the tibia was maintained and fixed using external fixation. The remaining wounds were covered with vacuum-sealed drainage (Wuhan Weiside Medical Technology Co., Ltd., Wuhan, China). In the second stage, skin grafting or flap transplantation was performed within 3–21 days after injury to cover the wound. Outpatient or ward treatments were provided for secondary wounds. In the third stage, the external fixation was removed after wound healing, and the plaster fixation was replaced with nail canal care. In the fourth stage, bone defect reconstruction and final fixation were performed without needle canal infection. Ilizarov bone transfer or internal fixation and autogenous cancellous bone grafting were performed. Details of the antibiotic regimen are provided in Supplemental Digital Content 1, available at: http://links.lww.com/JS9/E757.

**Orthoplastic approach**: This method was divided into three stages. In the first stage, after the patient was injured, the emergency doctor was prompted by the regional internet system to inject antibiotics intravenously as soon as possible (within 1 h of injury)^[9]^. The recommended regimen includes cefuroxime combined with aminoglycoside antibiotics, or clindamycin for patients with a β-lactam allergy. The emergency physician evaluated, photographed, rinsed, and bandaged the wounds. Once a dressing was in place, it was typically not removed until the patient reached the operating room. This prevented continuous blood loss and contamination. In the operating room, experienced doctors performed thorough debridement of the inactive and contaminated tissues. Completely free bone fragments were treated on a case-by-case basis, and the completely free cortical bone located in the metaphysis and shaft was removed. This is because the presence of free and inactive fragments greatly increases the risk of infection. Bone fragments that were connected to soft tissues and had blood supply were preserved. The “pull test” was used to determine the removal of bone fragments with a soft tissue adhesion rate below 50%^[18]^. Large bone fragments that were completely free were temporarily used for reduction and discarded at the end of debridement, whereas bone fragments with completely free epiphyseal trabecular bone and articular surfaces were retained and fixed with tension screws after thorough debridement and flushing (for a detailed description of the debridement procedure, see Supplemental Digital Content 2, available at: http://links.lww.com/JS9/E565). The remaining wounds were covered with vacuum-sealed drainage (Wuhan Weiside Medical Technology Co., Ltd., Wuhan, China). In cases classified as Gustilo type IIIC, we also repaired all anterior and posterior tibial arteries, which enabled us to prepare for the selection of suitable blood supply vessels during the second stage of skin flap transplantation and increased the chances of limb survival. The other options were the same as those used for the Gustilo type IIIB. External fixation was performed to maintain the tibial length. Postoperative intravenous cefuroxime combined with aminoglycoside antibiotics and clindamycin were administered to patients with β-lactam allergies. If the wound was contaminated with organic substances (including agricultural or forestry residues, aquatic elements from fisheries and waterways, substances related to animal husbandry, military industry byproducts, soil contaminants, or fecal matter), penicillin or metronidazole was used in combination with these antibiotics. Antibiotics were administered for 24 h after complete soft tissue coverage but discontinued after 72 h^[19]^. Details of the antibiotic regimen are provided in Supplemental Digital Content 1, available at: http://links.lww.com/JS9/E757.

In the second stage (postoperative days 1–7), preoperative computed tomography angiography was used to identify the perforating vessels of the anterior lateral thigh flap^[20]^. The external fixator was removed and replaced with an internal fixation (plate or intramedullary nail). At this stage, the damaged area was thoroughly debrided again, and the wound was rinsed with 9 L of isotonic sodium chloride solution. Some patients were implanted with a mixture of gentamicin and vancomycin bone cement in the bone-defect area^[21]^. With technological improvements, we attempted to use bone cement in some patients. Block molding implantation facilitated the removal of bone cement and reduced the risk of damage to the induction membrane during secondary removal. All wounds that required skin flap coverage were selected as free anterolateral thigh flaps. In the third stage (4-6 weeks after the end of the second stage), the bone cement was removed, and the defect was filled with autogenous cancellous bone. We created a three-dimensional tibial model using the Mimics software (version 21.0, Materialse Inc, Belgium) to accurately calculate the length and volume of each bone defect. The follow-up periods ranged from 26 to 62 months. We summarize this treatment plan as the Flap and Open Reduction Internal Fixation and Masquelet (FOM) technique.

**Figure 1.**
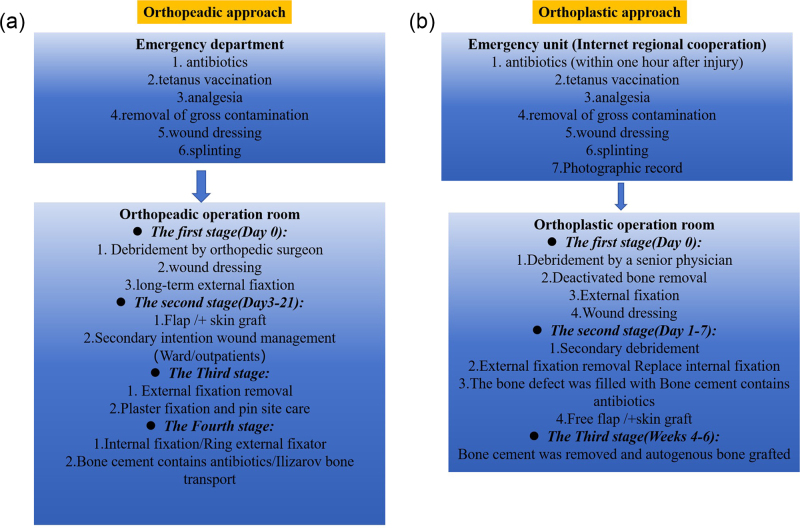
Comparison between simple orthopedic approach (a) and orthoplastic approach (b) of open tibial fractures.

The main treatment methods of the orthopedic and orthoplastic approaches used in this study are described in Figure 1a and b. In the orthopedic group, external fixation was used to treat fractures for a long time, and negative-pressure dressing was the most common strategy in this group. In the orthoplastic group, temporary external fixation was the conventional initial method, followed by intramedullary nail or plate replacement within 1–7 days. In the orthoplastic group, the free anterolateral thigh flap was the main support for wound coverage, and skin grafting was feasible for the remaining wounds with good bedding. The typical FOM technique is shown in Supplemental Digital Content 3, available at: http://links.lww.com/JS9/E566.

### Postoperative management and follow-up

Postoperative patients were housed in the orthopedic ward before December 2012, and after 2013, they were housed in the professional orthoplastic surgery ward, which included staff with expertise in graft and flap management and programmed nursing. All patients were followed-up, with the first follow-up starting in the second week after discharge, primarily focusing on wound healing and inflammatory indicators. Subsequently, follow-up was performed once a month for at least 26 months to ensure fracture healing (defined as full weight-bearing without pain and the presence of radiographic bone callus in two mutually perpendicular planes).

### General information and outcome assessments

To minimize information bias, standard data collection forms were obtained from the electronic equipment of the institution. Information regarding patient sex, age, smoking and drinking status, presence of diabetes mellitus, fracture classification (Gustilo**–**Anderson type IIIB/C), fracture location (proximal, middle, or distal), final fixation method (plate, intramedullary nail, or circular external fixation), whether antibiotic bone cement was locally used, whether bone cement was formed in blocks, whether reaming or non-reaming was selected in the intramedullary nail fixation method, and post-injury Mangled Extremity Severity score was collected. The volume of bone defect (volume of bone defect after final fixation and volume requiring bone grafting in the later stage) was measured using the Mimics software. The time of wound coverage (defined as the time required for complete repair of the soft tissue barrier at the fracture site) and the follow-up time were estimated.

### Outcome variables

The outcome variables were as follows: (1) deep infection, referred to as fracture-related infection: consensus of the international expert group definition^[22]^; (2) superficial infection (positive skin culture based on clinical suspicion); (3) nonunion of bone; (4) number of surgeries; (5) fracture healing time, defined as the time from final fixation to complete weight-bearing without pain and the presence of radiographic callus in two mutually perpendicular planes; (6) amputation; (7) arthritis; and (8) Lower Extremity Functional Scale (LEFS) scores, recorded at various intervals post-injury (2 weeks, 1 month, 3 months, 6 months, 12 months, and 24 months). This systematic data collection enabled a comprehensive analysis of functional recovery over time.

### Statistics

Continuous data are expressed as means ± standard deviations when conforming to a normal distribution; otherwise, they are expressed as medians and ranges. Normality was tested using the Kolmogorov–Smirnov test. The Wilcoxon rank-sum test or Kruskal–Wallis test was used to compare continuous variables between the groups. For comparisons of categorical variables, we used Fisher’s exact test for expected frequencies of <5; otherwise, we used the chi-square test. We hypothesized that the type of surgery, namely the orthopedic approach or the orthoplastic approach, influences the outcomes, independent of the surgeon, perioperative protocols (nutrition, anesthesia, and nursing), and the timing of the surgery. To improve the reliability of our findings and ensure their relevance to real-world clinical practice, we used propensity score matching and 1:1 best-matching algorithms to account for any changes in the baseline characteristics between the two treatment groups, thereby reducing selection bias and the impact of potential confounders. Propensity matching analysis was performed using standardized mean difference (SMD), which was calculated using the matchit package of the R software, with SMD >0.2 indicating an imbalance. To assess potential differences in the population, we examined the interactions between the orthopedic and orthoplastic approaches, and various predefined subgroups. A univariate logistic regression analysis was used to evaluate the relationship between each covariate and the outcome variable. A multivariate logistic regression analysis was used to determine independent factors significantly associated with clinical efficacy, and potential confounders were adjusted. All variables from the univariate analysis were included in the multivariate logistic regression model, regardless of their significance. This comprehensive method ensures that all potential influencing factors are considered and adjusted, and therefore that independent risk factors with a significant impact can be identified accurately. All statistical analyses were performed using the R software (version 4.2.2, RStudio Inc, USA). The level of significance was set at *P* < 0.05.

## Results

### Description of the study sample

This study included 746 patients: 420 in the orthopedic group and 326 in the orthoplastic group. The baseline characteristics of patients undergoing either the orthopedic surgery or the orthoplastic surgery were assessed across multiple variables (Table 1). The two groups were generally similar in terms of age, with mean ages of 77 ± 13 and 76 ± 13 years for the orthopedic and orthoplastic groups, respectively. Sex distribution was also comparable, with women constituting 41.7% and 39.0% of the orthopedic and orthoplastic groups, respectively, and men comprising 58.3% and 61.0%, respectively. Notably, a significant difference in smoking status was observed between the groups, with a higher percentage of smokers in the orthoplastic group (54.0%) than in the orthopedic group (44.3%) (*P =* 0.009). Drinking habits and diabetes prevalence were not significantly different between the groups. A greater prevalence of IIIB fractures according to the Gustilo–Anderson classification was observed in the orthoplastic group (87.7%) compared to the orthopedic group (75.0%). The Mangled Extremity Severity score, bone defect volume, and follow-up duration did not differ significantly between the groups. However, significant differences were observed in wound coverage time, which was notably shorter for the orthoplastic approach (mean of 4.0 ± 2.0 days) than for the orthopedic approach (12.1 ± 5.6 days) (*P* < 0.001). The fracture location varied, with the orthoplastic group having more midshaft fractures (51.5%) than the orthopedic group (39.3%) (*P* = 0.002). Fixation methods also differed significantly, with the orthoplastic group undergoing reconstruction with bone cement blocks considerably more often than the orthopedic group (85.9% vs. 18.8%, *P* < 0.001). Finally, intramedullary reaming was more common in the orthopedic group (84.4%) than in the orthoplastic group (66.2%) (*P* < 0.001), whereas the use of local antibiotics was similar in both groups.

**Table 1:** Patient demographics and baseline characteristics

Characteristic	Treatment		*P*-value
	Orthopedic approach, *N* = 420^a^	Orthoplastic approach, *N* = 326^a^	
Age			0.331^b^
Mean ± SD	56 ± 10	54 ± 10	
Median (IQR)	56(50, 62)	55 (47, 61)	
Range	30, 87	36, 79	
Sex			0.455^c^
Female	175 (41.7)	127 (39.0)	
Male	245 (58.3)	199 (61.0)	
Smoking			0.009^c^
Yes	186 (44.3)	176 (54.0)	
No	234 (55.7)	150 (46.0)	
Drinking			0.776^c^
Yes	194 (46.2)	154 (47.2)	
No	226 (53.8)	172 (52.8)	
Diabetes			0.785^c^
Yes	30 (7.1)	25 (7.7)	
No	236 (92.9)	301 (92.3)	
Gustilo–Anderson grade			<0.001^c^
IIIB	315 (75.0)	286 (87.7)	
IIIC	105 (25.0)	40 (12.3)	
MESS score			0.883^b^
Mean ± SD	9.54 ± 2.25	9.51 ± 2.28	
Median (IQR)	8.00 (5.00, 11.00)	9.00 (5.25, 12.00)	
Range	5.00, 13.00	5.00, 12.00	
Bone defect (cm3)			0.546^b^
Mean ± SD	10.6 ± 5.9	11.7 ± 7.5	
Median (IQR)	7.9 (6.5, 16.2)	7.9 (6.3, 17.7)	
Range	3.2, 28.0	1.9, 34.8	
Wound coverage time (days)			<0.001^b^
Mean ± SD	12.1 ± 5.6	4.0 ± 2.0	
Median (IQR)	12.0 (7.0, 17.0)	4.0 (2.0, 6.0)	
Range	3.0, 21.0	1.0, 7.0	
Fracture site			0.002^c^
Proximal	109 (26.0)	58 (17.8)	
Middle	165 (39.3)	168 (51.5)	
Distal	146 (34.8)	100 (30.7)	
Fixation			<0.001^c^
Plate	96 (22.9)	98 (30.1)	
IN	270 (64.3)	228 (69.9)	
CE	54 (12.9)	0 (0.0)	
Local antibiotics			0.870^c^
Yes	379 (90.2)	293 (89.9)	
No	41 (9.8)	33 (10.1)	
SBCM			<0.001^c^
Yes	79 (18.8)	280 (85.9)	
No	341 (81.2)	46 (14.1)	
IN reaming			<0.001^c^
Yes	228 (84.4)	151 (66.2)	
No	42 (15.6)	77 (33.8)	
Follow-up (months)			0.736^b^
Mean ± SD	54 ± 11	55 ± 10	
Median (IQR)	62 (47, 62)	62 (48, 62)	
Range	26, 62	26, 62	

IN = intramedullary nail, CE = circular external, SBCM = segmented bone cement molding.

^a^ Data are represented as *n* (%).

^b^ Wilcoxon rank-sum test.

^c^ Pearson’s chi-square test.

### Propensity matching analysis

After propensity score matching, the resulting cohort consisted of 326 patients each in the orthopedic and orthoplastic groups. All SMD values after matching were <0.10, indicating that the matching effect was good and that there were no significant differences in confounding factors between the two groups (Table 2).

**Table 2:** Baseline covariates before and after matching

Variables	Level	Before matching				After matching		
		Orthopedic approach	Orthoplastic approach	SMD		Orthopedic approach	Orthoplastic approach	SMD
N		420	326			326	326	
Age (mean (SD))		56.06 (10)	54 (10)	−0.078		58 (12.71)	60 (13.15)	0.006
Sex (%)	Female	175 (41.7)	127 (39.0)	−0.056		136 (41.7)	127 (39.0)	−0.057
	Male	245 (58.3)	199 (61.0)	0.056		190 (58.3)	199 (61.0)	0.007
Smoking (%)	Yes	186 (44.3)	176 (54.0)	0.195		147 (45.1)	176 (54.0)	0.008
	No	234 (55.7)	150 (46.0)	−0.195		179 (54.9)	150 (46.0)	−0.078
Drinking (%)	Yes	194 (46.2)	154 (47.2)	0.021		156 (47.9)	154 (47.2)	−0.012
	No	226 (53.8)	172 (52.8)	−0.021		170 (52.1)	172 (52.8)	−0.012
Diabetes (%)	Yes	30 (7.1)	25 (7.7)	0.093		25 (7.7)	28 (8.6)	0.002
	No	390 (92.9)	301 (92.3)	−0.093		301 (92.3)	298 (91.4)	−0.092
MESS score (mean (SD))		9.54 (2.25)	9.51 (2.28)	−0.012		9.51 (2.22)	9.51 (2.28)	0.001
Bone defect (cm^3^) (mean (SD))		10.57 (5.87)	11.69 (7.51)	−0.192		10.57 (5.87)	9.68 (5.40)	0.001
Wound coverage time (days) (mean (SD))		12.06 (5.58)	3.98 (1.98)	−4.083		11.27 (5.14)	3.98 (1.98)	0.004
Gustilo–Anderson grade (%)	IIIB	315 (75.0)	286 (87.7)	0.388		247 (75.8)	286 (87.7)	−0.005
	IIIC	105 (25.0)	40 (12.3)	−0.388		79 (24.2)	40 (12.3)	−0.065
Fracture site (%)	Proximal	109 (26.0)	58 (17.8)	−0.213		82 (25.2)	58 (17.8)	−0.092
	Middle	165 (39.3)	168 (51.5)	0.245		131 (40.2)	168 (51.5)	0.007
	Distal	146 (34.8)	100 (30.7)	−0.089		113 (34.7)	100 (30.7)	−0.086
Fixation (%)	Plate	96 (22.9)	98 (30.1)	0.157		86 (26.4)	98 (30.1)	−0.040
	IN	270 (64.3)	228 (69.9)	0.123		240 (73.6)	228 (69.9)	−0.080
	CE	54 (12.9)	0 (0.0)	−0.512		0 (0.0)	0 (0.0)	0.000
Local antibiotics (%)	Yes	379 (90.2)	293 (89.9)	−0.012		294 (90.2)	293 (89.9)	−0.010
	No	41 (9.8)	33 (10.1)	0.012		32 (9.8)	33 (10.1)	0.010
SBCM (%)	Yes	79 (18.8)	280 (85.9)	1.927		69 (21.2)	280 (85.9)	−0.059
	No	341 (81.2)	46 (14.1)	−1.927		257 (78.8)	46 (14.1)	−0.059
IN reaming (%)	Yes	228 (84.4)	151 (66.2)	−0.321		178 (74.2)	151 (66.2)	−0.054
	No	42 (15.6)	77 (33.8)	0.321		62 (25.8)	77 (33.8)	−0.054
Follow-up (months) (mean (SD))		54.15 (10.98)	54.94 (9.89)	0.080		54.56 (10.54)	54.94 (9.89)	0.008

IN = intramedullary nail, CE = circular external, SBCM = Segmented bone cement molding, SMD = standardized mean difference.

### Clinical outcomes

**Figure 2.**
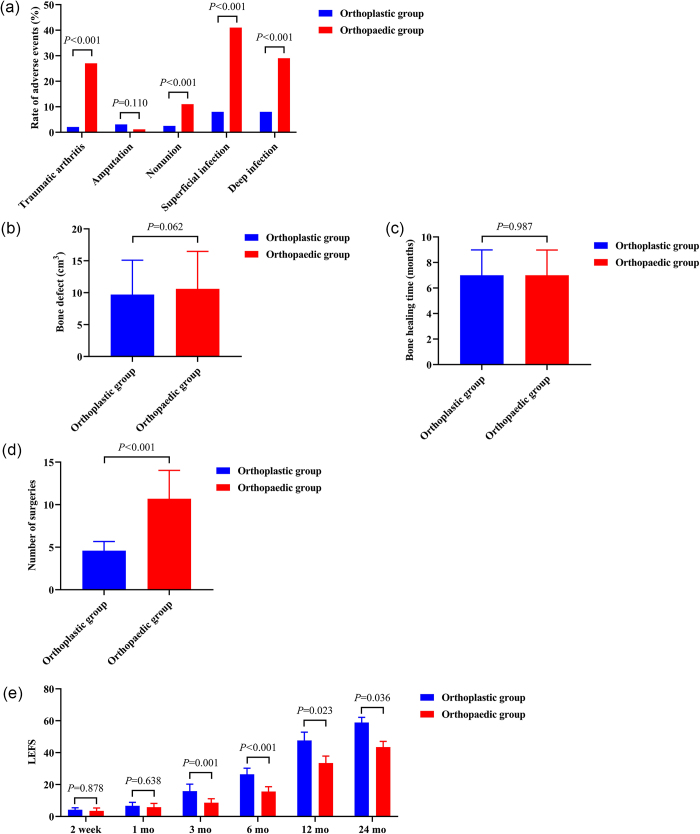
Clinical outcomes of orthoplastic and orthopedic groups.

The clinical results after matching, including the rates of deep and superficial infection, incidence of bone nonunion, number of surgeries, fracture healing time, amputation rate, incidence of arthritis, and LEFS scores after injury, are shown in Figure 2.

The infection rate in the orthoplastic group was significantly lower than that in the orthopedic group (deep infection, 8.0% vs. 29%; superficial infection, 8.0% vs. 41%; all *P* < 0.001). The incidence of nonunion was significantly higher in the orthopedic group than in the orthoplastic group (11% vs. 2.5%, *P* < 0.001). The incidence of arthritis was significantly higher in the orthopedic group than in the orthoplastic group (27% vs. 2.1%, *P* < 0.001). No significant difference in the amputation rate was observed between the two groups (1.2% vs. 3.1%, *P* = 0.11). After evaluation, two senior surgeons agreed to perform below-knee amputation in 8 patients from the orthoplastic group and 12 patients from the orthopedic group. They were amputated because of failure of the initial treatment attempt (severe compression of the limb, severe microvascular injury at the receiving site) or life-threatening injury.

No significant difference in fracture healing time was observed between the orthoplastic and orthopedic groups (7.0 ± 1.99 vs. 7.0 ± 1.98, *P* = 0.987). The number of surgeries required in the orthoplastic group was significantly lower than that in the orthopedic group (4.6 ± 1.07 vs. 10.7 ± 3.33, *P* < 0.001). There was no statistically significant difference in bone defect volume between the orthoplastic group and the orthopedic group (9.7 ± 5.39 cm^3^ vs. 10.6 ± 5.87 cm^3^, *P* = 0.062).

The results indicated no significant difference in LEFS scores between the orthoplastic and orthopedic groups at 2 weeks post-injury (4.2 ± 1.2 vs. 3.5 ± 1.8, *P* = 0.878). Similarly, at 1 month post-injury, no significant difference in LEFS scores was observed between the two groups (6.7 ± 2.1 vs. 5.8 ± 2.4, *P* = 0.638). However, at 3 months post-injury, the LEFS scores of the orthoplastic group (15.9 ± 4.3) were significantly higher than those of the orthopedic group (8.6 ± 2.5) (*P* = 0.001). At 6 months post-injury, the orthoplastic group exhibited higher LEFS scores (26.4 ± 3.8) than the orthopedic group (15.6 ± 3.0) (*P* < 0.001). At 12 months post-injury, the orthoplastic group maintained higher LEFS scores (47.6 ± 5.2) than the orthopedic group (33.5 ± 4.3) (*P* = 0.023). Finally, at 24 months post-injury, the LEFS scores of the orthoplastic group (58.9 ± 3.2) were significantly higher than those of the orthopedic group (43.5 ± 3.5) (*P* = 0.036).

### Subgroup analysis and interaction analysis of the effect of internal fixation methods on bone defect volume (bone graft volume)

**Figure 3.**
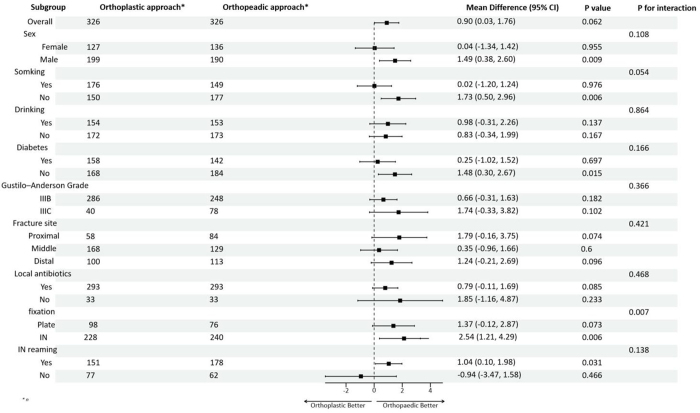
Subgroup analysis and interaction analysis of the effect of internal fixation methods on bone defect volume (bone graft volume).

No significant difference in the bone graft volume was observed between the orthoplastic and orthopedic groups (*P* = 0.062). The subgroup analysis showed that the results were not affected by sex, smoking, drinking, diabetes mellitus, Gustilo classification, fracture site, local antibiotics, or intramedullary nail reaming. However, heterogeneity was observed in the fixation method (interaction *P* = 0.007). The amount of bone graft in the orthopedic group was greater than that in the orthoplastic group (*P* = 0.073) when plate fixation was used, and the amount of bone graft in the orthopedic group was greater than that in the orthoplastic group *(P* = 0.006) when intramedullary nail fixation was used, indicating that the use of intramedullary nails in the orthoplastic group may reduce the amount of bone graft required (Fig. 3).

### Clinical outcomes of plate versus intramedullary nail in the orthoplastic group

To compare the efficacy and safety of intramedullary nail or plate fixation in the orthoplastic group, we analyzed 326 patients in the orthoplastic group separately and conducted a retrospective control study using intramedullary nails or plates. The baseline characteristics of the two groups are shown in Table 3. A significant difference was observed in age distribution, with the mean age of the intramedullary nail group (79 ± 11 years) being higher than that of the plate group (74 ± 14 years) (*P* = 0.008). No additional statistically significant differences in baseline characteristics were observed between the two groups.

**Table 3:** Orthoplastic patient demographics and baseline characteristics

Characteristic	Fixation		*P*-value
	Plate, *N* = 98^a^	IN, *N* = 228^a^	
Age, Mean (SD)	54 (11)	53 (14)	0.008^b^
Sex			0.431^c^
Male	63 (64.3)	136 (59.6)	
Female	35 (35.7)	92 (40.4)	
Smoking			0.344^c^
Yes	49 (50.0)	127 (55.7)	
No	49 (50.0)	101 (44.3)	
Drinking			0.167^c^
Yes	52 (53.1)	102 (44.7)	
No	46 (46.9)	126 (55.3)	
Diabetes			0.626^c^
Yes	13(13.3)	35 (15.4)	
No	85(86.7)	193 (84.6)	
MESS score			0.843^b^
Mean ± SD	9.47 ± 2.31	9.53 ± 2.27	
Median (IQR)	9.50 (5.00, 11.00)	10.00 (5.00, 12.00)	
Range	5.00, 12.00	5.00, 12.00	
Gustilo–Anderson grade			0.706^c^
IIIB	87 (88.8)	199 (87.3)	
IIIC	11 (11.2)	29 (12.7)	
Fracture site			0.271^c^
Proximal	22 (22.4)	36 (15.8)	
Middle	45 (45.9)	123 (53.9)	
Distal	31 (31.6)	69 (30.3)	
Local antibiotics			0.102^c^
Yes	84 (85.7)	209 (91.7)	
No	14 (14.3)	19 (8.3)	
SBCM			0.148^c^
Yes	80 (81.6)	200 (87.7)	
No	18 (18.4)	28 (12.3)	
IN reaming			0.744^c^
Yes	76 (77.6)	173 (75.9)	
No	22 (22.4)	55 (24.1)	
Wound coverage time (days)			0.367^b^
Mean ± SD	3.83 ± 2.02	4.04 ± 1.97	
Median (IQR)	4.00 (2.00, 6.00)	4.00 (2.00, 6.00)	
Range	1.00, 7.00	1.00, 7.00	
Follow-up (months)			0.658^b^
Mean ± SD	55 ± 10	55 ± 10	
Median (IQR)	62 (46, 62)	62 (49, 62)	
Range	29, 62	26, 62	

IN = intramedullary nail, SBCM = segmented bone cement molding.

^a^ Data are represented as *n* (%).

^b^ Wilcoxon rank-sum test.

^c^ Pearson’s chi-square test.

After propensity score matching, the resulting cohort consisted of 98 patients each in the intramedullary nail and orthoplastic groups. All SMD values after matching were <0.10, indicating that the matching effect was good and that there were no significant differences in confounding factors between the two groups (Table 4).

**Table 4:** Baseline covariates before and after matching

Variables	Level	Before matching				After matching		
		IN	Plate	SMD		IN	Plate	SMD
*n*		228	98			98	98	
Age (mean (SD))		53.92 (10)	54.89 (8.61)	0.113		53.27 (10.47)	54.89 (8.61)	0.188
Sex (%)	Male	136 (59.6)	63 (64.3)	0.097		63 (64.3)	63 (64.3)	0.000
	Female	92 (40.4)	35 (35.7)	−0.097		35 (35.7)	35 (35.7)	0.000
Smoking (%)	Yes	127 (55.7)	49 (50.0)	−0.114		49 (50.0)	49 (50.0)	0.000
	No	101 (44.3)	49 (50.0)	0.114		49 (50.0)	49 (50.0)	0.000
Drinking (%)	Yes	102 (44.7)	52 (53.1)	0.167		49 (50.0)	52 (53.1)	0.061
	No	126 (55.3)	46 (46.9)	−0.167		49 (50.0)	46 (46.9)	−0.061
Diabetes (%)	Yes	118 (51.8)	40 (40.8)	−0.223		35 (35.7)	40 (40.8)	−0.104
	No	110 (48.2)	58 (59.2)	0.223		63 (64.3)	58 (59.2)	−0.104
Gustilo–Anderson grade (%)	IIIB	199 (87.3)	87 (88.8)	0.047		89 (90.8)	87 (88.8)	−0.065
	IIIC	29 (12.7)	11 (11.2)	−0.047		9 (9.2)	11 (11.2)	0.065
Fracture site (%)	Proximal	36 (15.8)	22 (22.4)	0.160		19 (19.4)	22 (22.4)	0.073
	Middle	123 (53.9)	45 (45.9)	−0.161		49 (50.0)	45 (45.9)	−0.082
	Distal	69 (30.3)	31 (31.6)	0.029		30 (30.6)	31 (31.6)	0.022
Local antibiotics (%)	Yes	209 (91.7)	84 (85.7)	−0.170		86 (87.8)	84 (85.7)	−0.058
	No	19 (8.3)	14 (14.3)	0.170		12 (12.2)	14 (14.3)	0.058
SBCM (%)	Yes	200 (87.7)	80 (81.6)	−0.157		79 (80.6)	80 (81.6)	0.026
	No	28 (12.3)	18 (18.4)	0.157		19 (19.4)	18 (18.4)	−0.026
IN reaming (%)	Yes	173 (75.9)	76 (77.6)	0.040		76 (77.6)	76 (77.6)	0.000
	No	55 (24.1)	22 (22.4)	−0.040		22 (22.4)	22 (22.4)	0.000
MESS score (mean (SD))		9.53 (2.27)	9.47 (2.31)	−0.025		9.28 (2.36)	9.47 (2.31)	0.084
Wound coverage time (days) (mean (SD))		4.04 (1.97)	3.83 (2.02)	−0.106		3.85 (2.00)	3.83 (2.02)	−0.010
Follow-up (months) (mean (SD))		55.08 (10.02)	54.61 (9.64)	−0.048		55.55 (9.98)	54.61 (9.64)	−0.097

IN = intramedullary nail, SBCM = segmented bone cement molding, SMD = standardized mean difference.

**Figure 4.**
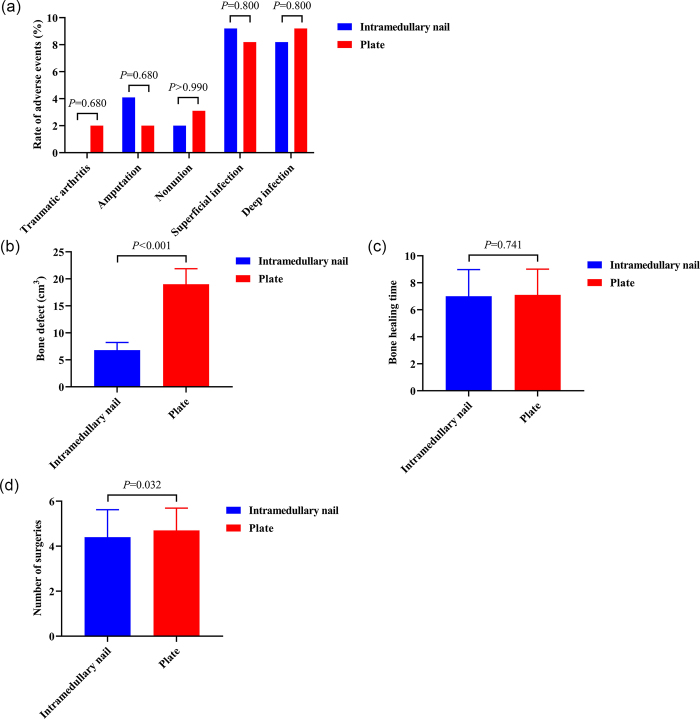
Clinical outcomes of plate versus intramedullary nail in the orthoplastic group.

The results showed no significant difference in the infection rates between the two groups (deep infection, 8.2% vs. 9.2%; superficial infection, 9.2% vs. 8.2%; all *P* = 0.80). No significant difference in the incidence of nonunion was observed between the two groups (2% vs. 3.1%, *P* > 0.99). No significant difference in the incidence of arthritis was observed between the two groups (0% vs. 2%, *P* = 0.68). No significant difference in the amputation rate was observed between the two groups (4.1% vs. 2.0%, *P* = 0.68), and no significant difference in fracture healing time was observed between the orthoplastic and orthopedic groups (7.0 ± 1.98 vs. 7.1 ± 1.91, *P* = 0.741). The number of operations in the intramedullary nail group was slightly less than that in the plate group (4.4 ± 1.22 vs. 4.7 ± 0.99, *P* = 0.032). We also used Mimics software to measure the volume of bone defect, and found that the amount of bone graft required in the intramedullary nail group was significantly less than that required in the plate group (6.8 ± 1.42 cm^3^ vs. 19.0 ± 2.88 cm^3^, *P* < 0.001) (Fig. 4).

### Impact of local antibiotic use vs. nonuse on superficial and deep infections in the orthoplastic group

To compare the impact of local antibiotic use on deep and superficial infections within the orthoplastic group, we conducted a retrospective controlled study involving 326 patients categorized according to the use of local antibiotics. The baseline characteristics of both groups are presented in Table 5. A significant difference was observed in the time to wound coverage, with the group not treated with local antibiotics exhibiting a shorter average time to wound coverage (3.15 ± 1.75 years) than the group treated with antibiotic bone cement (4.07 ± 1.99 years) (*P* = 0.008). No additional statistically significant differences were found in the baseline characteristics between the two groups.

**Table 5:** Patient demographics and baseline characteristics

Characteristic	Local antibiotics		*P*-value
	Yes	No	
	*N* = 293^a^	*N* = 33^a^	
Age	76 ± 13	76 ± 13	0.981^b^
Sex			0.485^c^
Male	177 (60.4)	22 (66.7)	
Female	116 (39.6)	11 (33.3)	
Smoking			0.764^c^
Yes	159 (54.3)	17 (51.5)	
No	134 (45.7)	16 (48.5)	
Drinking			0.210^c^
Yes	135 (46.1)	19 (57.6)	
No	158 (53.9)	14 (42.4)	
Diabetes			0.587^c^
Yes	26 (8.8)	2 (6.0)	
No	267 (91.2)	31(93.9)	
Gustilo–Anderson Grade			0.577^d^
IIIB	258 (88.1)	28 (84.8)	
IIIC	35 (11.9)	5 (15.2)	
Fixation			0.102^c^
Plate	84 (28.7)	14 (42.4)	
IN	209 (71.3)	19 (57.6)	
Fracture site			0.224^c^
Proximal	49 (16.7)	9 (27.3)	
Middle	155 (52.9)	13 (39.4)	
Distal	89 (30.4)	11 (33.3)	
IN reaming			0.101^c^
Yes	220 (75.1)	29 (87.9)	
No	73 (24.9)	4 (12.1)	
MESS	9.51 ± 2.26	9.48 ± 2.46	0.952^b^
Duration of wound coverage (days)	4.07 ± 1.99	3.15 ± 1.75	0.008^b^
Follow-up (months)	55 ± 10	56 ± 9	0.649^b^
SBCM			0.597^d^
Yes	250 (85.3)	30 (90.9)	
No	43 (14.7)	3 (9.1)	

IN = intramedullary nail, SBCM = segmented bone cement molding.

^a^ Data are represented as Mean ± SD; *n* (%).

^b^ Welch’s two-sample *t*-test.

^c^ Pearson’s chi-squared test.

^d^ Fisher’s exact test.

Following the application of propensity score matching, the resulting cohort comprised 33 patients who received local antibiotic treatment and 33 patients who did not. All SMD values were found to be <0.10 after matching, indicating a high quality of matching and the absence of significant confounding factor differences between the two patient groups (Table 6).

**Table 6:** Baseline covariates before and after matching

Variables	Level	Before matching				After matching		
		Yes	No	SMD		Yes	No	SMD
*n*		293	33			33	33	
Age (mean (SD))		75.85 (13.21)	75.91 (12.83)	0.004		71.12 (14.95)	75.91 (12.83)	0.073
Sex (%)	Male	177 (60.4)	22 (66.7)	0.133		21 (63.6)	22 (66.7)	0.064
	Female	116 (39.6)	11 (33.3)	−0.133		12 (36.4)	11 (33.3)	−0.064
Smoking (%)	Yes	159 (54.3)	17 (51.5)	−0.055		15 (45.5)	17 (51.5)	0.021
	No	134 (45.7)	16 (48.5)	0.055		18 (54.5)	16 (48.5)	−0.021
Drinking (%)	Yes	135 (46.1)	19 (57.6)	0.233		15 (45.5)	19 (57.6)	0.045
	No	158 (53.9)	14 (42.4)	−0.233		18 (54.5)	14 (42.4)	−0.045
Diabetes (%)	Yes	26 (8.8%)	2 (6.0%)	−0.067		10 (30.3)	2 (6.00)	0.004
	No	267 (91.2%)	31(93.9%)	0.067		23 (69.7)	31 (93.9)	−0.004
Gustilo–Anderson Grade (%)	IIIB	258 (88.1)	28 (84.8)	−0.089		28 (84.8)	28 (84.8)	0.000
	IIIC	35 (11.9)	5 (15.2)	0.089		5 (15.2)	5 (15.2)	0.000
Fracture site (%)	Proximal	49 (16.7)	9 (27.3)	0.237		9 (27.3)	9 (27.3)	0.000
	Middle	155 (52.9)	13 (39.4)	−0.276		10 (30.3)	13 (39.4)	0.086
	Distal	89 (30.4)	11 (33.3)	0.063		14 (42.4)	11 (33.3)	−0.093
Fixation (%)	Plate	84 (28.7)	14 (42.4)	0.278		12 (36.4)	14 (42.4)	0.023
	IN	209 (71.3)	19 (57.6)	−0.278		21 (63.6)	19 (57.6)	−0.023
SBCM (%)	Yes	250 (85.3)	30 (90.9)	0.194		32 (97.0)	30 (90.9)	−0.011
	No	43 (14.7)	3 (9.1)	−0.194		1 (3.0)	3 (9.1)	0.011
IN reaming (%)	Yes	220 (75.1)	29 (87.9)	0.392		28 (84.8)	29 (87.9)	0.093
	No	73 (24.9)	4 (12.1)	−0.392		5 (15.2)	4 (12.1)	−0.093
MESS (mean (SD))		9.51 (2.26)	9.48 (2.46)	−0.011		9.48 (2.20)	9.48 (2.46)	0.000
Duration of wound coverage (mean (SD))		4.07 (1.99)	3.15 (1.75)	−0.523		3.03 (2.10)	3.15 (1.75)	0.069

SMD = standardized mean difference IN = intramedullary nail, SBCM = segmented bone cement molding.

The results indicated a significant difference in the infection rates between the two groups. For deep infections, the rate was 6.1% in the group treated with local antibiotics and 24% in the non-treated group (*P* = 0.039). The rate of superficial infections was 6.1% in the group treated with local antibiotics and 18.0% in the non-treated group (*P* = 0.026).

### Risk factors for nonunion

To identify the potential risk factors for nonunion in the orthoplastic group, we first performed a univariate logistic regression analysis to evaluate the association between individual factors (sex, smoking and drinking status, presence of diabetes mellitus, Gustilo classification, fracture site, local antibiotic use, bone cement block molding, and intramedullary nail reaming) and the outcome variable “nonunion.” Nonsmokers had a lower risk of bone nonunion than smokers (odds ratio [OR], 0.28; 95% confidence interval [CI], 2.29–4.79; *P* = 0.019). The risk of fracture nonunion without bone cement block molding was higher than that with it (OR, 1.25; 95% CI, 2.10–7.21; *P* = 0.005). Intramedullary nail without reaming had a higher risk of nonunion than intramedullary nail reaming (OR, 1.45; 95% CI, 2.06–3.76; *P* = 0.045) (Table 7).

**Table 7:** Univariate and multivariate analyses affecting nonunion (logistic regression)

Characteristic	Univariable			Multivariable			Multiple imputation		
	OR^a^	95% CI^a^	*P*-value	OR1	95% CI^a^	*P*-value	OR^a^	95% CI^a^	*P*-value
Sex									
Male	—	—		—	—		—	—	
Female	0.94	0.22, 4.00	0.932	0.66	0.15, 2.96	0.589	0.66	0.15, 2.98	0.589
Smoking									
Yes	—	—		—	—		—	—	
No	0.28	0.29, 0.47	0.019	0.24	0.22, 0.34	0.008	0.24	0.29, 0.37	0.008
Drinking									
Yes	—	—		—	—		—	—	
No	1.51	0.35, 6.41	0.579	1.64	0.37, 7.32	0.520	1.64	0.36, 7.37	0.520
Diabetes									
Yes	—	—		—	—		—	—	
No	0.56	0.13, 2.37	0.427	0.69	0.15, 3.09	0.627	0.69	0.15, 3.11	0.628
Gustilo–Anderson grade									
IIIB	—	—		—	—		—	—	
IIIC	0.00	0.00, 8.35	0.992	0.00	0.00, 9.22	0.997	0.00	0.00, 9.22	0.997
Fracture site									
Proximal	—	—		—	—		—	—	
Middle	3.79 × 10^06^	0.00, 4.26	0.991	2.56 × 10^07^	0.00, 5.88	0.996	2.56 × 10^07^	0.00, 5.88	0.996
Distal	2.01 × 10^07^	0.00, 4.87	0.990	1.54 × 10^08^	0.00, 6.50	0.996	1.54 × 10^08^	0.00, 6.50	0.996
Local antibiotics									
Yes	—	—		—	—		—	—	
No	0.00	0.00, 2.66	0.993	0.00	0.00, 3.20	0.997	0.00	0.00, 3.20	0.997
SBCM									
Yes	—	—		—	—		—	—	
No	1.25	2.10, 7.21	0.005	1.54	2.06, 4.74	0.007	1.54	2.06, 4.78	0.007
IN reaming									
Yes	—	—		—	—		—	—	
No	1.45	2.06, 3.76	0.045	0.40	0.05, 3.47	0.403	0.40	0.04, 3.50	0.403

^a^ OR = odds ratio, CI = confidence interval, IN = intramedullary nail, SBCM = segmented bone cement molding.

nimp = 5.00; No. Obs. = 326.

To identify independent factors significantly associated with “nonunion” while adjusting for potential confounders, a multivariate logistic regression analysis was performed. Nonsmokers had a lower risk of bone nonunion than smokers (OR, 0.24; 95% CI, 2.29–5.34; *P* = 0.008). The risk of fracture nonunion without bone cement block molding was higher than that with it (OR, 1.54; 95% CI, 2.06–4.73; *P* = 0.007) (Table 7).

### Risk factors for deep infection

The univariate logistic regression analysis showed that nonsmokers had a lower risk of deep infection than smokers (OR, 0.33; 95% CI, 0.13–0.83; *P* = 0.019). The risk of deep infection was higher in middle fractures than in proximal fractures (OR, 1.23; 95% CI, 1.39–3.89; *P* = 0.028). Nonuse of local antibiotics increased the risk of deep infection (OR, 4.89; 95% CI, 1.93–12.37; *P* < 0.001). Therefore, smoking, middle-segment fractures, and nonuse of local antibiotics were identified as potential risk factors for deep infections (Table 8).

**Table 8:** Univariate and multivariate analysis affecting deep infection (logistic regression)

Characteristic	Univariable			Multivariable			Multiple imputation		
	OR^a^	95% CI^a^	*P*-value	OR^a^	95% CI^a^	*P*-value	OR^a^	95% CI^a^	*P*-value
Sex									
Male	—	—		—	—		—	—	
Female	0.35	0.13, 1.95	0.089	0.47	0.16, 1.35	0.160	0.47	0.16, 1.36	0.161
Smoking									
Yes	—	—		—	—		—	—	
No	0.33	0.13, 0.83	0.019	0.26	0.10, 0.71	0.009	0.26	0.09, 0.72	0.009
Drinking									
Yes	—	—		—	—		—	—	
No	0.37	0.16, 2.87	0.073	0.33	0.13, 2.85	0.071	0.33	0.13, 2.85	0.071
Diabetes									
Yes	—	—		—	—		—	—	
No	0.94	0.42, 2.08	0.870	0.99	0.41, 2.38	0.986	0.99	0.41, 2.38	0.986
Gustilo–Anderson grade									
IIIB	—	—		—	—		—	—	
IIIC	0.93	0.27, 3.24	0.906	0.87	0.22, 3.40	0.842	0.87	0.22, 3.42	0.843
Fracture site									
Proximal	—	—		—	—		—	—	
Middle	1.23	1.39, 3.89	0.028	1.45	0.44, 4.86	0.542	1.45	0.43, 4.88	0.543
Distal	1.17	0.34, 4.08	0.801	1.63	0.44, 6.10	0.469	1.63	0.43, 6.13	0.469
Fixation									
Plate	—	—		—	—		—	—	
IN	0.80	0.34, 1.85	0.598	0.99	0.39, 2.53	0.985	0.99	0.39, 2.54	0.985
Local antibiotics									
Yes	—	—		—	—		—	—	
No	4.89	1.93, 12.37	<0.001	4.79	1.70, 13.49	0.003	4.79	1.70, 13.54	0.003
SBCM									
Yes	—	—		—	—		—	—	
No	0.23	0.03, 1.71	0.151	0.23	0.03, 1.84	0.166	0.23	0.03, 1.86	0.167
IN reaming									
Yes	—	—		—	—		—	—	
No	0.75	0.27, 2.07	0.584	1.08	0.36, 3.25	0.892	1.08	0.36, 3.26	0.892
Duration of wound coverage									
≤72 h	—	—		—	—		—	—	
72 h–3 days	1.30	0.25, 6.84	0.756	1.16	0.21, 6.42	0.866	1.16	0.21, 6.42	0.866

^a^ OR = odds ratio, CI = confidence interval.

nimp = 5.00; No. Obs. = 326.

The multivariate logistic retrospective analysis showed that nonsmokers had a lower risk of deep infection than smokers (OR, 0.26; 95% CI, 0.10–0.71; *P* = 0.009). Patients in whom local antibiotics were not used had a high risk of deep infection (OR, 4.89; 95% CI, 1.93–12.37; *P*<0.001). Therefore, smoking and nonuse of local antibiotics were identified as independent risk factors for deep infections (Table 8).

The univariate and multivariate logistic retrospective analyses suggested that the use of plates and intramedullary nails had no significant effect on deep infections (OR, 0.99; 95% CI, 0.39–2.53; *P* = 0.985) (Table 8).

## Discussion

### Advantages of the orthoplastic approach

Open tibial fractures, particularly Gustilo–Anderson type IIIB/C fractures, represent a prevalent form of orthopedic trauma that poses substantial challenges in clinical orthopedic management owing to extensive soft tissue damage and bone exposure. This complexity leads to increased treatment difficulties and a high incidence of complications. Traditional orthopedic surgery adheres to a staged protocol comprising “debridement, external fixation, delayed soft tissue management, and definitive fixation.”^[23]^ This approach prioritizes the stabilization of the fracture site through external fixation and negative pressure wound therapy, while postponing flap surgery. Although the initial technical difficulty is relatively low, the delay in soft tissue coverage frequently leads to increased infection rates. Prolonged external fixation can also result in complications such as pin tract infections and joint stiffness. Research indicates that the pin tract infection rate associated with external fixation can reach 17.2%^[24]^, while the incidence of joint stiffness can be as high as 38%^[25].^ Furthermore, inadequate biomechanical stability often leads to delayed union.

Orthoplastic surgery integrates orthopedic reconstruction with the principles of plastic surgery, achieving simultaneous fracture fixation, soft tissue defect repair, and wound closure in a single-stage combined procedure. This approach employs flap transplantation techniques, which reduce the risk of infection and accelerate functional recovery. In 1986, Godina *et al* proposed the application of microsurgical reconstruction in cases of complex limb trauma, underscoring the need for prompt soft tissue coverage^[26]^. Naique *et al* further advocated the integration of orthopedic and plastic surgical interventions for the management of severe open tibial fractures within specialized centers^[27].^ The British Orthopaedic Association and British Association of Plastic Surgeons have also published relevant guidelines, emphasizing the critical need for early transfer of patients to specialized centers for orthoplastic reconstruction^[28]^. The findings of this study indicate that the orthoplastic approach significantly outperformed the orthopedic approach in terms of infection rate, nonunion incidence, arthritis incidence, number of surgeries, and wound coverage time, although no significant difference in fracture healing time was observed between the two groups. These results indicate that the orthoplastic approach offers significant advantages in the treatment of Gustilo–Anderson type IIIB/C open tibial fractures. The infection rate in this cohort was lower than that reported in international studies, in which the deep infection rate at the fracture site was 9.5%^[11]^. Although there was no statistically significant difference in the LEFS scores at 2 weeks and 1 month post-injury, significant differences emerged at 3, 6, 12, and 24 months post-injury, with the orthoplastic group demonstrating superior outcomes compared to those of the orthopedic group. This suggests that implementation of the orthoplastic approach facilitates early functional recovery in these patients.

### Comparison of clinical outcomes between plate and intramedullary nail

Gustilo**–**Anderson type IIIB/C open tibial fractures are frequently associated with significant soft tissue contamination and defects. Historically, internal fixation has been largely avoided because of concerns regarding infection risk, biofilm formation, and potential damage to the vascular supply^[29]^. However, advancements in debridement techniques to ensure a sterile environment have led to the increasing popularity and acceptance of internal fixation. In such cases, appropriate internal fixation can facilitate improved healing and functional recovery. Gopal *et al* introduced the concept of “Fix and Flap,” wherein orthoplastic approaches were employed to simultaneously stabilize and cover wounds in emergency situations involving Gustilo**–**Anderson type IIIB/C open tibial fractures^[11]^. Among the patients studied, 19 received external fixation devices for fracture stabilization, and 65 underwent internal fixation. The findings indicated that compared with external fixation, internal fixation was associated with a low infection rate, accelerated fracture healing, and few complications. In this study, we present the concept of the FOM technique. Among the 326 patients treated with the orthoplastic approach, external fixation was utilized solely during the emergency phase, with all patients subsequently transitioning to internal fixation (intramedullary nail or plate) within 1–7 days. Concurrently, soft tissue coverage was completed, and the Masquelet technique was employed to address critical-sized bone defects.

The use of plates or intramedullary nails remains controversial, and most clinical studies do not favor the application of plates. Van der Linden and Larsson conducted a randomized controlled trial comparing plate fixation and conservative treatment in 100 patients^[30]^. Their findings indicated that the healing time in the plate-treated group for open fractures was nearly twice as high compared to that in the conservative treatment group. Notably, only two patients in the plate-treated group experienced no complications. The study design mandated wound healing before surgical intervention. Similarly, Bach and Hansen conducted a randomized clinical trial involving 59 patients with type II and III open tibial fractures and randomly assigned them to either an external fixation group using half pins or an internal plate fixation group. The results showed that the plate fixation group exhibited higher rates of wound infection (35% vs. 13%), chronic osteomyelitis (19% vs. 3%), and internal fixation failure (12% vs. 7%) than the external fixation group. While the external fixation group had a lower rate of pin-tract infection (10%), it also had a higher rate of malunion than the internal fixation group (10% vs. 4%)^[31]^ Clifford *et al* conducted a non-comparative chart review of 97 patients with fractures fixed with plates, 60 of whom had type II or III open fractures. Their report noted a deep tissue infection rate of 10.3% among these patients^[32]^, with nearly half (44%) of these deep tissue infections occurring after plate fixation in type III open fractures. This phenomenon may be attributed to the inert surface of the plate, which creates a conducive environment for bacterial growth. Consequently, the ongoing controversy regarding the use of plates largely centers on the potential for chronic infection and the subsequent risk of infected nonunion.

The use of intramedullary nails minimizes further damage to soft tissues and the periosteum while allowing patients to bear weight early postoperatively, thereby preventing a range of complications associated with prolonged bed rest. Furthermore, because the surgical incision and insertion point of the intramedullary nail are located away from the open wound, bacterial contact and proliferation are significantly reduced. Giovannini *et al* conducted a meta-analysis comparing the effectiveness of intramedullary nailing and external fixation for the treatment of Gustilo type III open tibial shaft fractures^[33].^ The findings indicated that compared with external fixation, intramedullary nailing significantly decreased the incidence of infection and issues related to fracture healing, with no significant differences observed in other complications. Liu *et al* conducted a meta-analysis to compare the efficacy and safety of external fixators and intramedullary nails in the treatment of open tibial fractures^[34]^. The results indicated that intramedullary nails significantly reduced the rates of postoperative superficial infection and malunion while also resulting in short fracture healing times. Furthermore, the use of intramedullary nails did not increase the risk of deep infections. In this study, we compared the outcomes of the intramedullary nails and plates in the orthoplastic group. The findings revealed no significant difference in the infection rates between the two groups. However, the intramedullary nail group required less amount of bone grafting in the later stages than the plate group. Traditional methods for assessing bone defects typically use the defect length as an evaluation criterion. However, bone defects are fundamentally a three-dimensional spatial issue. In this study, we employed the Mimics software for the first time to accurately assess the volume of bone defects. Notably, our assessment time point was selected after the final internal fixation surgery rather than immediately after injury. This approach enables precise prediction and calculation of the bone volume required for subsequent bone-grafting surgeries, thereby enhancing the precision and effectiveness of the treatment. Previous studies have seldom addressed this aspect.

### Risk factors and preventive measures for deep infections

The prevention of infection relies on several critical factors. Notably, the low deep infection rate (8.0%) observed in the orthoplastic approach group may be attributed to the early administration of systemic antibiotics, thorough debridement, skeletal stabilization within 1–7 days, simultaneous coverage of the wound with a free anterolateral thigh flap, and implementation of the Masquelet technique-Get it Right First Time Principle. The specific antibiotic regimens and debridement methods used in the orthoplastic approach are detailed in Supplemental Digital Content 2, available at: http://links.lww.com/JS9/E565. In our orthoplastic series, we exclusively employed a free anterolateral thigh flap for flap selection, which is commonly referred to as the “universal flap.” While local flaps, such as regional muscle flaps, can be used to cover small defects, patients with Gustilo type IIIB/C open tibial fractures often experience significant local muscle and soft tissue damage. Based on previous experiences, the survival rate of local flaps is typically lower than that of free flaps.

There is a consensus on the early use of systemic antibiotics after open fractures; however, the use of local antibiotics remains controversial. The application of local antibiotics, such as antibiotic-loaded polymethylmethacrylate bone cement beads that can steadily release antibiotics at the target site, has garnered widespread attention in recent years. A retrospective study conducted by Ostermann *et al* involving 1085 patients with open fractures showed that the combination of local application of tobramycin-impregnated bone cement beads and systemic antibiotic prophylaxis significantly reduced the rates of acute and chronic infections in patients with type III open fractures compared with systemic antibiotic prophylaxis alone (6.5% vs. 20.6%, respectively)^[33]^. A meta-analysis of 21 studies indicated that the combined use of local and systemic antibiotics significantly reduced the risk of deep infections in patients with open fractures of the lower extremities^[35]^. This study involved various types of open tibial fractures treated with intramedullary nailing, with the most pronounced effect observed in type III open fractures. The combination of local and systemic antibiotics demonstrated a more significant effect in preventing infections than the use of systemic antibiotics alone. We found that most infections had significant predisposing factors; the nonuse of local antibiotics (OR, 4.89; 95% CI, 1.93–12.37; *P* < 0.001) was identified as an independent risk factor for deep infections. To further validate the role of local antibiotics in infection prevention, we compared the impact of local antibiotic use and nonuse on the infection rates in patients undergoing orthoplastic surgery. The results revealed that the application of local antibiotics significantly reduced the rates of both deep and superficial infections in Gustilo type IIIB/C open tibial fractures, which is consistent with the findings of the infection risk factor analysis. Therefore, local antibiotic administration should be combined with systemic antibiotic use in patients with Gustilo type IIIB/C open tibial fractures. Specifically, it is advised to add 2–5 g of vancomycin to 40 g gentamicin bone cement powder (containing 0.5 g gentamicin)^[36]^.

### Strengths and limitations of the study

The strengths of our study include the inclusion of 746 patients with Gustilo**–**Anderson type IIIB/C open tibial fractures, which improved the statistical power of our results. Data were prospectively collected from the hospital database to reduce information and recall biases. The propensity matching method balanced the baseline characteristics of the two patient groups, reduced selection bias, and improved the reliability of the causal inference. In addition, the follow-up time in this study was 26–62 months, which can be used to evaluate the long-term efficacy and complications. In contrast to the situation in other countries, many orthopedic doctors in China not only have the skills and expertise for fracture fixation but also those for microsurgery, including free flap transplantation, which helps reduce the heterogeneity of surgical effects caused by different surgeons. We propose the FOM scheme, which provides a new approach for the treatment of open tibial fractures.

This study has some limitations. Although the propensity matching method was used, there are still potential confounding factors in retrospective studies that cannot completely rule out selection bias. In this study, we included covariates that have been previously reported in the literature as potentially influencing prognosis. During the data collection process, we made every effort to comprehensively gather various clinical variables and relevant patient information to explore these potential factors in subsequent analyses. We recognize that a variety of factors must be comprehensively considered in the research process regarding their impact on prognosis, rather than solely focusing on differences in surgical approaches. We hypothesize that the type of surgery – specifically, the orthopedic approach versus the orthoplastic approach – may influence outcomes independently of the surgeon, perioperative protocols (including nutrition, anesthesia, and nursing), and the timing of surgery. These variables were not included in the study. The single-center design of the study helped to reduce the differences in observations between observers; however, the results may not be generalizable to other hospitals or regions. The reliability of the results of the subgroup analysis requires further verification, and large-scale multicenter studies are needed.

## Conclusion

The orthoplastic approach was significantly better than the orthopedic approach in terms of the infection rate, incidence of nonunion, and incidence of arthritis. There was no significant difference in fracture healing time between the two groups. Smoking should be avoided when undergoing the orthoplastic approach, and bone cement block molding technology should be actively used to reduce the risk of fracture nonunion. Systemic and local antibiotics should be administered as early as possible to reduce the risk of deep infection. When adopting the FOM technique, an intramedullary nail is a better choice than a plate because it reduces the amount of bone graft required without increasing the risk of deep infection. This scheme optimizes the treatment process and provides a new approach for the treatment of Gustilo**–**Anderson type IIIB/C open tibial fractures.

## Data Availability

The data that support the findings of this study are available fromthe corresponding author upon reasonable request.
